# Agricultural productivity in relation to climate and cropland management in West Africa

**DOI:** 10.1038/s41598-020-59943-y

**Published:** 2020-02-25

**Authors:** Altaaf Mechiche-Alami, Abdulhakim M. Abdi

**Affiliations:** 1 Department of Physical Geography and Ecosystem Science, Lund University, SE-223 62 Lund, Sweden; 2 Centre for Environmental and Climate Research, Lund University, SE-223 62 Lund, Sweden

**Keywords:** Attribution, Ecosystem services

## Abstract

The climate of West Africa is expected to become more arid due to increased temperature and uncertain rainfall regimes, while its population is expected to grow faster than the rest of the world. As such, increased demand for food will likely coincide with declines in agricultural production in a region where severe undernutrition already occurs. Here, we attempt to discriminate between the impacts of climate and other factors (e.g. land management/degradation) on crop production across West Africa using satellite remote sensing. We identify trends in the land surface phenology and climate of West African croplands between 2000 and 2018. Using the combination of a an attribution framework and residual trend anlaysis, we discriminate between climate and other impacts on crop productivity. The combined effect of rainfall, land surface temperature and solar radiation explains approximately 40% of the variation in cropland productivity over West Africa at the 95% significance level. The largest proportions of croplands with greening trends were observed in Mali, Niger and Burkina Faso, and the largest proportions with browning trends were in Nigeria, The Gambia and Benin. Climate was responsible for 52% of the greening trends and 25% of the browning trends. Within the other driving factors, changes in phenology explained 18% of the greening and 37% of the browning trends across the region, the use of inputs and irrigation explained 30% of the greening trends and land degradation 38% of the browning trends. These findings have implications for adaptation policies as we map out areas in need of improved land management practices and those where it has proven to be successful.

## Introduction

Over the last 100 years, average near-surface temperatures in Africa have increased by 0.5 °C^1,2^. Projected increases in temperature exceeding average temperature variability experienced in the twentieth century are expected to occur 10 to 20 years earlier in Africa, particularly the Sahelian and tropical regions of West Africa, compared to the rest of the world^1,3^. Consequently, the climate of West Africa is expected to gradually become more arid as humid zones recede mostly due to temperature forcing, although variability in local rainfall is also important^4^.

These projected changes in climate will occur in addition to the intra- and inter-annual variability of rainfall that has historically caused extreme droughts and floods^5^. As such, both the projected trends and climatic variability pose a challenge for rainfed agriculture, which forms the foundation of food security, fulfilling 80% of the needs of the population and employing about 60% of the workforce in West Africa^6–8^. These challenges include changes in the start and length of growing seasons, harvest success, and subsequently agricultural production^6,9^. In West Africa, the main staple crops such as maize, cassava, millet, and sorghum are mostly dependent on rainfall^10^. The region is influenced by large-scale climate teleconnections^11^ and some of the largest deficits in crop production have been due to droughts induced by declines in rainfall^12^. Current estimates of changes in yield across West Africa vary between studies. However, there is general agreement that yield reduction could reach as low as −41% with +1.5 °C warming^13–16^.

The human population of West Africa is growing faster than the rest of the world. Of the projected increase in global population between 2015 and 2050, roughly 60%, or 1.3 billion people, will be in Africa. This corresponds to an increase of 30% in the population of West Africa^17^. Furthermore, a large proportion of agricultural products is appropriated by the population of the region^18^. Thus, increased demand for food, feed, fuel, and forage in the future^19^ will likely coincide with declines in agricultural production^16^. In view of these future risks, West African countries adopted the Regional Agricultural Policy for West Africa in 2005 based on technology-sharing to increase agricultural production as well as a more integrated agricultural market^8^. They also implemented the West African Agricultural Productivity Program in 2007 to improve crop production and smallholder farmers’ incomes through investment programs focusing on water and soil management as well as input dissemination^8,20^. Finally, in 2015 the West African Alliance for Climate Smart Agriculture (CSA) was adopted to integrate adaptation measures within a sustainable agricultural system^20^. In view of the political will to adopt policies that would increase crop productivity in a sustainable manner, assessing the relationship between climate change, land-use and their impact on crop growth is essential^2^.

Land surface phenology (LSP), observed using satellite remote sensing, represents the seasonal variability of terrestrial vegetation and is used to quantify the timing and duration of the phenological phases of vegetation^21^. Vegetation phenology including emergence, maturity, and senescence can be detected across large areas with Earth-observing satellites using proxies such as start of the growing season, length of the growing season, peak greenness and accumulated biomass^21^. LSP is an efficient indicator for monitoring the response of terrestrial ecosystems to changes in climate because of the wide spatial coverage provided by satellite observations and can thus provide necessary data for crop modeling^21–24^. Several studies have already mapped LSP across Africa, especially along the Sahel, and related the trend in LSP mostly to changes in rainfall and soil moisture^25–33^. However, few studies have focused on assessing productivity and LSP trends of croplands to isolate climatic and other impacts in these managed lands. Moreover, earlier studies are mostly performed at a relatively coarse resolution, e.g. pixel-size greater than 1 km, and fail to capture complexities that occur at finer scales^34^.

In this study, we focus on recent trends in vegetation productivity and LSP of West African croplands (Supplementary Fig. 1) and their relationship to changes in climate over a 19-year period spanning 2000 to 2018 at a spatial resolution of 250 m. We identify trends in the LSP parameters of start-of-season, length-of-season and vegetation productivity, which is quantified as the normalized difference vegetation index (NDVI) integrated over the growing season. We then evaluate the relationship between the integrated NDVI (iNDVI) and change in land surface temperature, rainfall, and solar radiation over the countries that constitute the Economic Community of West African States (ECOWAS). Finally, we discriminate between trends caused by climatic and other factors at a relatively fine resolution. The aim is to identify areas where adaptation measures have successfully circumvented negative climatic impacts, as well as areas still in need of localized adaptation that could be implemented towards improving food production.

## Results

### Trends in phenology and iNDVI

**Figure 1:**
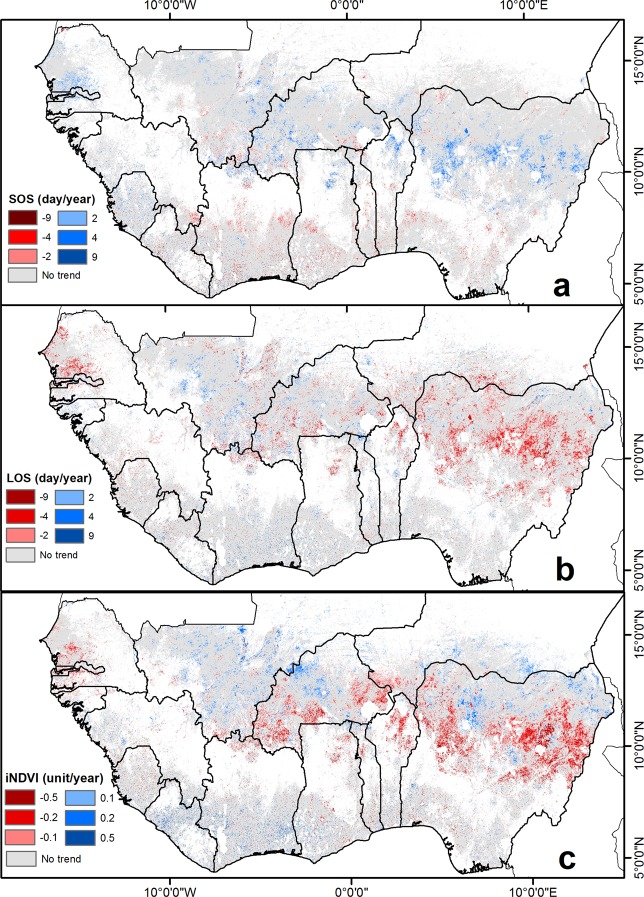
Trends in seasonality and integrated NDVI (2000–2018). Statistically significant trends (*P* < 0.05) for (**a**) start-of-season (SOS), (**b**) length-of-season (LOS) and (**c**) integrated normalized difference vegetation index (iNDVI).

We found statistically significant (*P* < 0.05) trends in SOS in 7% of ECOWAS croplands with delays observed along the Sahel as well as Sierra Leone and early onsets in southern Mali and the more humid zones of West Africa (Fig. 1a). Significant trends in LOS occur over 9% of croplands between 2000 and 2018 (Fig. 1b). Increases in LOS were observed in southern Mali and the coastal parts of Ghana and Côte d’Ivoire. Decreases in LOS were visible on both sides of the border between Niger and Nigeria as well as north central Burkina Faso and across Senegal.

The trend in iNDVI, which indicates a change in agricultural productivity over time, showed that around 15% of all the cropland pixels in the ECOWAS region had significant trends (Fig. 1c). Most increases in iNDVI were observed over Mali, central Burkina Faso, southern Niger, parts of northern Nigeria, as well as in Liberia, and the southern parts of Côte d’Ivoire, Ghana and Benin. On the other hand, negative iNDVI trends, indicating decreases in crop productivity, were observed in the Black Volta basin of the southern half of Burkina Faso and over large parts of Nigeria and northern Benin (Fig. 1c). The western coast of the study area also exhibited negative iNDVI trends, particularly in parts of Senegal, the Gambia, Guinea-Bissau, Guinea and Sierra Leone.

### Residual trend analysis

**Figure 2:**
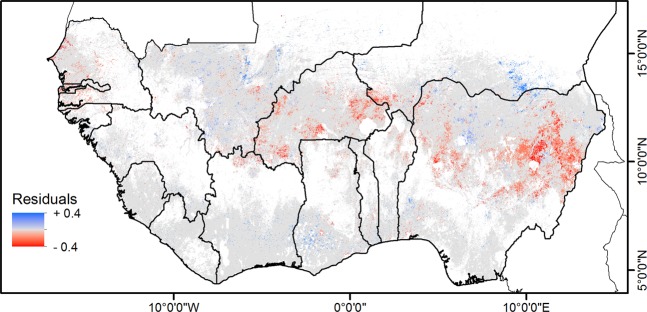
Residual trends (2000–2018). Statistically significant trend (*P* < 0.05) of the residuals from the relationship between integrated normalized different vegetation index (iNDVI) and climate (rainfall, land surface temperature and solar radiation).

The residual trend analysis, which shows the trend in iNDVI not explained by climate, resulted in positive trends in southern Mali, Niger, Côte d’Ivoire and Ghana (Fig. 2). Parts of northwestern Nigeria also exhibited positive trends in both the residuals and iNDVI (Fig. 1c). Negative residual trends were found over Senegal, The Gambia and Guinea-Bissau as well as most of Burkina Faso and large parts of Nigeria. Partial correlations between climate variables (rainfall, land surface temperature, and solar radiation) and iNDVI showed a stronger influence of rainfall with over 13% of the croplands having a positive correlation over the Sahel and negative correlations in Liberia and Sierra Leone. It is followed by land surface temperature (8%) with strong negative correlations observed in Mali, Burkina Faso and Niger and positive correlations in northern Nigeria and Liberia. Finally, solar radiation has a stronger influence on the iNDVI variability in 7% of the croplands, with mostly positive correlations in the humid zones and strongly negative correlations in Mali, Niger and Nigeria (Supplementary Fig. 2c). Altogether, the combined effect of the climatic variables significantly (*P* < 0.05) explains iNDVI variation in approximately 40% of the croplands in West Africa.

### Drivers of the trends

**Figure 3:**
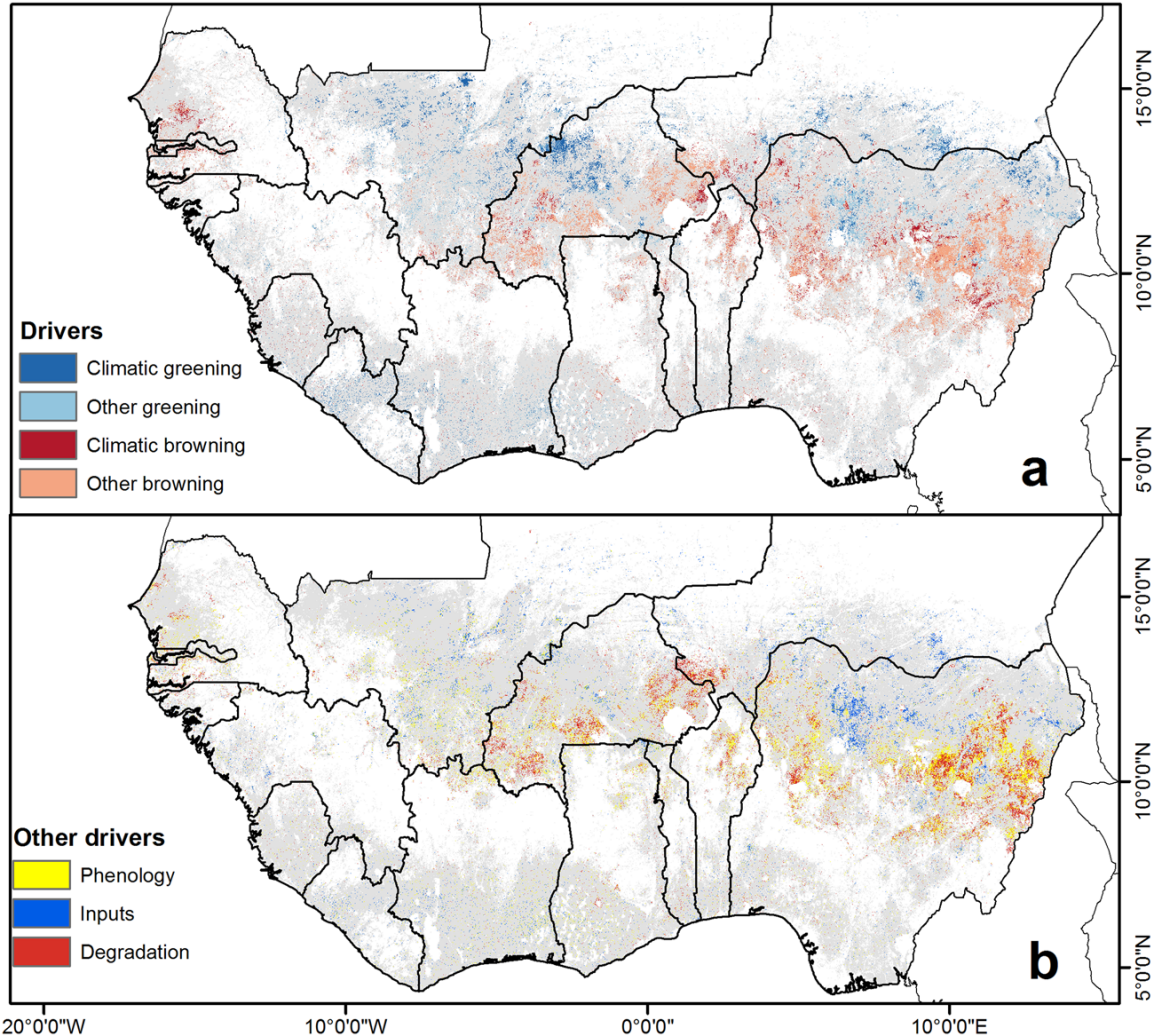
Drivers of vegetation change in West African croplands (2000–2018). This is based on the attribution framework detailed in the methods. The maps show (**a**) attribution of the drivers whether they are due to climate or other factors, and (**b**) designation of the other drivers considering the relationship between iNDVI and phenology.

**Figure 4:**
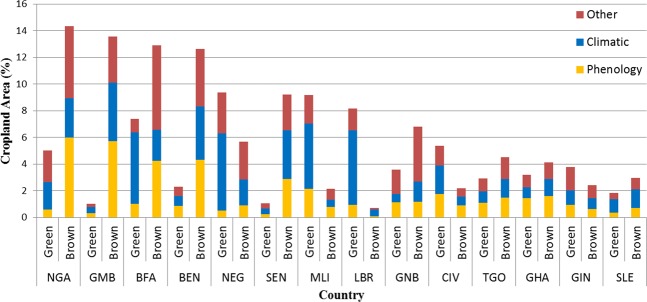
Attribution of iNDVI directional synergy between 2000 and 2018 by country. The total area of covered by greening or browning, according to the attribution framework, is dissected into its driving factors. This identifies change in cropland iNDVI in each country as being driven by climatic (rainfall, land surface temperature, and solar radiation), phenological (length-of-season), or other (inputs such as fertilizers or irrigation, or land degradation).

Recent changes in climatic variables were only responsible for 36% of the directional synergy (see methods) in significant iNDVI trends over West Africa. They were mostly linked to greening trends (21%) mostly in Mali and Burkina Faso, and browning trends (15%) in Senegal, Burkina Faso and Nigeria (Fig. 3a). The remaining trends were due to other factors with greening trends (20%) observed in southern Mali, across the Niger-Nigeria border and along the southern coast of West Africa and browning (44%) concentrated in Burkina Faso, Senegal, Nigeria and northern Benin (Fig. 3a). These other factors are attributed to changes in phenology (29%) throughout Burkina Faso and Nigeria, increased inputs (irrigation and fertilizers) in Mali and northwestern Nigeria as well as Côte d’Ivoire and Ghana, and to land degradation (23%) across Burkina Faso, Nigeria and Sierra Leone (Fig. 3b). Overall, regardless of attribution, the largest proportions of croplands with greening trends were observed in Mali, Niger and Burkina Faso (10.6%, 9.3% and 9.2%, respectively), and the largest proportions with browning trends were in Nigeria, The Gambia and Benin (14.3%, 13.6% and 13.5%, respectively) (Fig. 4).

## Discussion

Some of the strongest relationships between iNDVI and rainfall were found across the Sahelian part of the study area (Supplementary Fig. 2a) as vegetation sensitivity to rainfall increases with environmental aridity^35^. In most of the sub-humid and humid areas, iNDVI responded weakly to climate with all climatic variables showing low correlations except for solar radiation over the tree crop farming system. This could be explained by the fact that these regions have relatively even temperature throughout the year as well as sufficient rainfall^29^. That said, variables such as ozone^36^ and CO_2_ fertilization^37^ were not considered in this study and could influence iNDVI. Another explanation for the low impact of climate on iNDVI could also stem from the fact that NDVI saturates in densely vegetated areas, which is the case for this region^21^.

The analyzed cropland pixels in West Africa showed a slight net browning trend between 2000 and 2018 (Figs. 1c and 3). The spatial extent of the negative trends shown in Fig. 1c extends to the Black Volta basin and corroborates the findings of Le, *et al*.^38^ who found large-scale land degradation and concluded that around 65% of the entire Volta river basin had undergone human-induced degradation of vegetation productivity. In another study of the Black Volta basin, Nyamekye, *et al*.^39^ found that approximately 18% of the natural vegetation in the area was converted into agriculture and non-vegetated surfaces from 1999 to 2011, and this activity was the primary driver of the negative trends they observed.

There were also negative trends in iNDVI over large parts of Nigeria (Fig. 1c) consistent with the findings of Luan, *et al*.^40^ who found a clear distribution of significant negative trends at the 90% level between 2001 and 2010. There has been a dramatic reduction in indigenous plant species due to ongoing land degradation and an increase of non-native and drought-tolerant plants that were introduced through afforestation programs^41^. These regions have also exhibited increasing dryness and drought-like conditions^42^ resulting in later planting dates (i.e. delayed SOS), shorter LOS and leading to reductions in biomass (Fig. 1a). Finally, land degradation in large parts of Nigeria has been linked to intensive agriculture in combination with deforestation of woodlands and soil erosion^43^.

There were positive trends in iNDVI and the residuals (Figs. 1c and 2) around the northern Nigerian city of Kano, which we attributed to a mixture of climatic and other factors (Fig. 3). This part of the country has witnessed an increase in tree density to support the growing population’s (Supplementary Fig. 3) reliance on fuelwood as a source of household energy^44^. Just 200 kilometers north of Kano, in the Zinder region of Niger, the parklands of the Magaria – Boune cluster show distinct positive trends in iNDVI and residuals. This region has also witnessed a transformative increase in tree cover due to farmer-managed natural regeneration (FMNR) programs resulting in an average on-farm tree cover of 4–6%^45^. Positive trends in both the iNDVI and the residuals were also found around the southern parts of Liberia, Côte d’Ivoire, and Ghana, which suggest an enhancement in seasonal crop productivity driven mostly by increases in land surface temperature (Supplementary Fig. 4) and increased inputs (Fig. 3b) in the more humid parts. Similar patterns were also found by Mueller, *et al*.^46^ who characterized the southern coast of West Africa as an “ecoregional extreme” for NDVI increase driven primarily by land management. Similarly, the positive residual trends in parts of Burkina Faso are also the product of both increases in rainfall and land rehabilitation efforts such as stone bunds, agroforestry and mulching^28,39^. The greening trends were also attributed to the expansion of irrigation by Boschetti, *et al*.^47^.

The trends across a considerable portion of the Sudano-Sahelian belt are driven by phenology changes (Fig. 3b). These could be indirectly related to climate with changes in rainfall and temperature extending or shortening the LOS, or later rains causing delays in SOS. However, these trends could also be considered as adaptation to warmer temperatures with earlier planting used to avoid critical stages of crop growth coinciding with extreme temperatures, or they could be representative of shifts in cultivar or crop types.

The population of ECOWAS countries has been growing steadily since 2000, particularly surrounding urban hubs in Senegal, Mali, Burkina Faso, Niger, Benin, Ghana and Nigeria (Supplementary Fig. 3). Future projections estimate that Nigeria – which already hosts the only megacity in West Africa (Lagos) – alongside China and India will together account for 35% of the increase in the world’s urban population by 2050. Niger and Nigeria will also witness the sharpest increase in rural population alongside Ethiopia and Uganda^48^. The impact of population on land resources is often presented as a net negative effect but the situation in West Africa is more complex than that. On the one hand, population has caused increased pressure on land and contributed to soil degradation^49^. Degradation was observed across western Senegal, The Gambia, and Sierra Leone as well as in southern Burkina Faso, western Niger, across Nigeria and in northern Benin (Fig. 3b). On the other hand, there are ongoing land rehabilitation efforts^39,50^ and conservation and management of plant species^51^ to sustain a growing population^52^. We find evidence of improved cropping practices in southern Mali and across the southern coast of Liberia, Côte d’Ivoire and Ghana where other greening trends are observed. Côte d’Ivoire and Liberia have made agriculture the foundation of post-conflict recovery through farming campaigns and investment plans^53,54^. In Mali and Ghana major restructuring of the agricultural sector has been made through the expansion of irrigation^55^ and input subsidies and mechanization to modernize the sector^56,57^.

Projected increases in extreme weather event frequency and intensity put West Africa at the center of risk exposure in Africa by the mid-21^st^ century^58^. Most of the Sahelian belt of West Africa is already exposed to climatic extremes such as long periods of heat waves, with the most vulnerable areas located in Niger and Burkina Faso^58^. This highly exposed area is expected to extend towards the coast by 2050 increasing the vulnerability of more areas in Mali and northern Nigeria but also Guinea and Sierra Leone^58^. Land degradation was also significant in the southern humid areas of Ghana, Togo, Benin and Nigeria (Fig. 3b) that benefit from a more stable climate with sufficient rainfall and soil moisture. Humid and fertile regions experience inbound migration from more arid and degraded regions because they are regarded as unspoiled by land degradation and consequently become ignored by land rehabilitation initiatives^59^. These areas could become even more vulnerable to high climatic variation and more extreme rainfall events leading to heavy flooding, and should not be ignored by climate adaptation and land rehabilitation projects. Indeed, they should be prioritized for climate-resilient agricultural development as they face risks posed by future climate change. Moreover, the influence of Boko Haram in Northern Nigeria, and violent groups elsewhere in the region, more recently along the Burkina Faso-Niger border, cannot be ignored and could explain declines in crop productivity in those parts of the region as people migrate to safer regions^60^.

### Limitations

The use of annual rainfall and mean land surface temperature and solar radiation rather than restricting these variables to the growing season could add uncertainties from biophysical phenomena that occur in the dry season. While the growing season in the semi-arid Sahel is only limited to the months in which there is rainfall, it is not the case in the more humid zones of the southern part of the study area where two cropping seasons are possible. Moreover, total rainfall is not the only factor contributing to crop growth, as the distribution of rainfall is also important as well as the storage capacity of soils and consequently soil moisture. Similarly, crops are more sensitive to strong variations in temperature rather than average temperature as prolonged periods of high temperature during critical stages of crop development can lead to crop failure. These aspects are indicative of the non-linear relationship between climate and vegetation and possible interaction among climate variables, which makes quantifying the full impacts of climate on iNDVI challenging, and introduces uncertainty into the trend attribution^61^. Furthermore, certain limiting factors such as nutrient deposition and ozone concentrations as well as the CO_2_ fertilization effect^37,61^ were not included in this study. Other limitations include the use of LSP over humid zones, specifically cloud contamination and the diversity of vegetation with varying canopy heights^21^. Finally, the results presented in this study are subject to the accuracy of the land cover map and the consistency of pixels classified as croplands.

## Conclusion

In this study, we evaluated trends in LOS, SOS and iNDVI between 2000 and 2018 across West Africa. We found that these trends are consistent with each other as pixels with longer LOS exhibit earlier SOS and increased iNDVI. We identified increases in crop productivity along the Sahel, especially in Mali, and Niger, but also in Liberia, Côte d’Ivoire and Guinea, while decreases were observed in Senegal, The Gambia, Burkina Faso and over Nigeria. We established a spatially-explicit relationship between iNDVI and land surface temperature, solar radiation and total annual rainfall. This helped identify a stronger influence of climate on iNDVI in the arid and semi-arid regions compared to the sub-humid and humid zones. Moreover, we established a decision-based model focused on iNDVI and residual trends to discriminate between climatic and other impacts on crop growth. Croplands in the Sahel are not only sensitive to climatic changes (e.g. Mali, Senegal) but also to a large extent to other factors (e.g. Burkina Faso, Nigeria).

Finally, by controlling for the impact of phenological changes, we identified areas undergoing land degradation that leads to decreased crop productivity especially in Nigeria and Burkina Faso and Benin, and those areas where enhanced management practices are improving crop production in southern Mali, Niger and around Kano in Nigeria. As the ECOWAS countries are moving towards a common agricultural policy, these cases deserve special attention in order to promote land management practices that could be disseminated across the entire region.

## Data and Methods

### Study area

The study area encompasses member countries of the Economic Community of West African States (ECOWAS) with the exception of Cabo Verde (Supplementary Fig. 1). This area spans across four aridity zones: arid, semi-arid, sub-humid and humid, and is characterized by a variety of farming systems from agro-pastoral millet/sorghum to tree crop systems^62–64^.

### Data

Surface solar radiation downwards (hereafter solar radiation) and land surface temperature products used in the analysis were taken from the European Centre for Medium-Range Weather Forecasts (ECMWF) ERA5 reanalysis product with a spatial resolution of approximately 10 km × 10 km from 1 January 2000 to 31 December 2018 (Dee *et al*. 2011). The solar radiation dataset includes both direct and diffuse radiation and is equivalent to what is measured by a global pyranometer at the land surface^65^. Generally, air temperature is used to quantify optimum conditions for vegetation growth^66^ and assess the impact of climate warming. We used land surface temperature because it is linked to the exchange of energy between the vegetation canopy and the atmosphere. Declines in vegetation productivity due to high surface temperature could be the result of stomatal closure due to loss of moisture from the vegetation canopy^67^. Thus, a decrease in the latent heat flux will be met with a corresponding increase in the sensible heat flux, which manifests as the land surface temperature signal to the satellite sensor^68^. The land surface temperature data is referred to as land skin temperature in ERA5 terminology and represents temperature at the interface between the land surface and the atmosphere. The rainfall product used was the Climate Hazards Group InfraRed Precipitation with Station data (CHIRPS), which is a combination of satellite observations and weather station data at approximately 5 km × 5 km spatial resolution over the study period^69^.

Vegetation data were derived from the MODerate resolution Imaging Spectroradiometer (MODIS). The latest Collection 6 of MOD09Q1 surface reflectance data for the red (RED) and infrared (NIR) bands were downloaded at 250 m spatial and 8-day temporal resolutions between 2000 and 2018^70^. This data product provides atmospherically-corrected surface reflectance and was selected due to its consistent temporal extensibility back to the year 2000. The images consist of eight MODIS tiles that cover the vegetated region from Senegal to Nigeria and include all ECOWAS countries except for Cabo Verde. As the first available MODIS images are from 26 February, the first 7 images of the year were replaced by that same image to facilitate time series analysis. Due to a sensor malfunction, there is no image available for 17 June 2001 and was, therefore, replaced by the average of the previous and next image. Crop cover data was obtained from the ESA CCI land cover^71^ product at 300 m resolution for 2000 and 2018. Pixels that remained cropland in both dates were taken to establish a crop mask. All data were resampled to 250 m using a bilinear interpolation to match the resolution of the MODIS data.

NDVI was computed as the difference between the red and near infrared bands divided by their sum^72,73^. This vegetation index is widely used to estimate vegetation or land surface phenology over large areas and its responses to climatic or human perturbations^27,28,30,32^. Data from two quality files (*sur_refl_state_250m* and *sur_refl_qc_250m*) were extracted and used to classify pixel quality into good, average and poor, mostly related to the level of atmospheric correction and cloud contamination^70^ (Supplementary Table 1). Details of this procedure are available in the supplementary information.

### Time series analysis and phenology extraction

TIMESAT^74^ was used to extract vegetation phenology from the NDVI time series. TIMESAT employs a variety of functions in order to fit and smoothen the NDVI time series. We used an asymmetric Gaussian and double logistic functions as they have been proven to be more robust and better able to represent general phenological parameters than local filtering methods^75^. The quality files were used as additional noise reducing weights of 1 for good pixels, 0.8 for average pixels and 0 for bad pixels.

First, the study area was divided into 30 zones representing the intersection of agro-ecological zones^64^ and farming systems^63^ as vegetation indices are dependent on biomes^21^. The average NDVI for each zone was calculated for each 8-day period. Then, the best fitting function and its parameter set was chosen by inspecting the averaged time series in TIMESAT thereby reducing the 30 zones to 5 TIMESAT zones (Supplementary Fig. 6). These functions were then applied in order to process the time series on a pixel-by-pixel basis and derive phenological metrics for each growing season. The final phenological metrics are (1) start-of-season (SOS) – defined as the 8-day period when NDVI exceeds 20% of the ascending amplitude, (2) length-of-season (LOS) – defined as the difference between SOS and the end-of-season, and (3) iNDVI – defined as NDVI integrated over LOS but above the mean minimum NDVI outside the growing season. This final parameter captures the seasonal cyclic part of the vegetation signal and is a robust indicator of aboveground net primary production^76^.

### Trend analysis of phenological metrics

For each year, pixels where LOS was shorter than one month were considered anomalous and removed from further analysis as no crop has such a short growing cycle. In areas with two growing seasons, the LOS and iNDVI of both seasons were added. The trends in SOS, LOS and iNDVI were calculated for each pixel based on the rank-based non-parametric Theil-Sen (TS) estimate of slope^77,78^. The TS slope is the median of slopes computed between all possible pairwise values with *n* independent and dependent samples such that (*n* × (*n*−1)/2). This approach is similar to a bootstrapping whereby all possible pairwise combinations of samples are taken into consideration. TS slopes were also computed for the land surface temperature, rainfall and solar radiation. Only trends at the 95% level (*P* < 0.05) were included in further analysis.

### iNDVI – climate residual trends

In order to estimate climate change impacts on crop production, most crop models account for rainfall, temperature, solar radiation and CO_2_, while climate change is usually expressed in terms of change in rainfall and temperature as a result of changes in CO_2_ concentrations^1,34,79,80^. As such, we focus on establishing the pixel-wise relationship between crop iNDVI, land surface temperature, solar radiation and total annual precipitation using partial correlations^81^. This analysis partitions the variation in crop growth between that which is explained by rainfall independent of, and jointly with, land surface temperature and solar radiation. In order to account for potential non-linearity in the relationship we use a logarithmic relationship between iNDVI and the climate variables in a multiple regression. However, we did not consider the interaction between the climatic variables in order to avoid overfitting and producing spurious effects as the study period creates a relatively small temporal sample size (n = 19) for such analysis^82^.

Once the climate impact on the iNDVI of croplands was isolated, any residual change in vegetation can be explained by other factors such as enhanced land management or land degradation. This was done by employing the residual trend (RESTREND) method^83^, which is a pixel-based approach that detects trends of residuals based on the relationship between iNDVI (as a proxy of cropland productivity) and climate data. The residual component of this relationship, which is the difference between observed iNDVI and the iNDVI predicted by the multiple regression model, was calculated. The residuals represent changes in iNDVI that are not directly due to climate. As such, positive residual trends in agricultural areas can potentially indicate that crop productivity increases due to improvements in land management, irrigation or crop diversification while negative trends are more indicative of land degradation, possibly due to intensive agriculture^84^. Only residual trends at the 95% level (*P* < 0.05) were included in further analysis.

### Attribution framework to identify drivers of iNDVI trends

**Figure 5:**
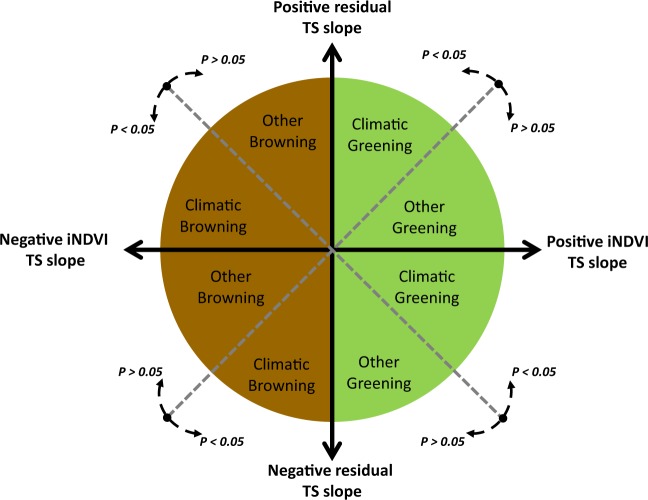
Conceptual framework based on directional synergy between iNDVI and residual trends. Decision rules of the framework to attribute greening and browning to climatic or other factors are based on the directional synergy between the Theil-Sen (TS) slope of iNDVI and the residuals resulting from the relationship between iNDVI and climate (rainfall, land surface temperature and solar radiation), which indicates change in iNDVI not driven by climate. Detailed notes of this figure are presented in the methods and in Supplementary Table 2.

In order to discriminate between the impacts of climatic and other factors on iNDVI, we built a set of decision rules to attribute greening or browning trends to either climate or other factors (Fig. 5). This was done following a modification of Leroux, *et al*.^31^ that uses the significance of the climate – iNDVI relationship and the direction of both iNDVI and residual trend slopes, i.e. the directional synergy between them at the 95% level (*P* < 0.05) (see Supplementary Table 2 for definitions). Therefore, pixels exhibiting a statistically significant positive iNDVI-climate relationship with a positive residual trend and a positive iNDVI trend suggests that both climate and other factors help increase vegetation productivity. Thus, if the iNDVI-climate relationship is non-significant, then we assume that other factors have suppressed the coupling between iNDVI and climate regardless of the sign of the iNDVI and residual trends. We then further elaborate on the nature of these factors based on the relationship between iNDVI and LOS^85^. Where this relationship was significant, iNDVI trends were attributed to changes in LOS while if the residuals of iNDVI and LOS were significantly positive, they were attributed to irrigation or fertilizer use and labeled “inputs”, and negative residual trends to land degradation.

## Supplementary information

Supplementary Information
