# Supplementary material for: Agricultural productivity in relation to climate and cropland management in West Africa
Source: Sci Rep. 2020 Feb 25;10:3393. doi: 10.1038/s41598-020-59943-y (PMC7042338; doi:10.1038/s41598-020-59943-y)
Supplement: Supplementary file 1 — Supplementary Information [file 41598_2020_59943_MOESM1_ESM.docx]

**Supplementary Information:**

**Agricultural productivity in relation to climate and cropland management in West Africa**

Altaaf Mechiche-Alami ^1,*^, Abdulhakim M. Abdi ^2, 3^

^1^ Department of Physical Geography and Ecosystem Science, Lund University, SE-223 62 Lund, Sweden

^2^ Centre for Environmental and Climate Research, Lund University, SE-223 62 Lund, Sweden

^3^ Department of Geosciences and Natural Resource Management, University of Copenhagen, DK-1350 Copenhagen, Denmark

* Corresponding author: altaaf.mechiche-alami@nateko.lu.se

**Data Quality**

Data from two quality files (sur_refl_state_250m and sur_refl_qc_250m) was extracted and used as to classify pixels into good, average and bad ^[[1]](#footnote-2)^. From the state flags, bits 0-1, 2, 3-5, and 13 were extracted and from the quality control, bits 0-1 and 12 were extracted. The good category consists of pixels clear from clouds (bits 0-1 of state flag), without cloud shadow (bit 2 from state flag), only on land (bits 3-5 from state flag), not adjacent to a cloud (bit 13 from state flag), ideal quality for both red and near-infrared bands (bits 0-1 from quality control) and the atmospheric correction should have been performed (bit 12 from quality control). A pixel is considered bad if the pixel is cloudy (bits 0-1 of state flag), the pixel does not cover land (bits 3-5 from state flag), the overall quality of both bands is not ideal (bits 0-1 from quality control) or the atmospheric correction was not performed (bit 12 from quality control). Any other bit combination results in an average pixel.

**Supplementary Table 1.** Criteria used to assess pixel quality of each MODIS image

|  | **Bit** | **Description** | **Good (AND)** | **Bad (OR)** |
| --- | --- | --- | --- | --- |
| **State Flag** | 0-1 | Cloud state | 0 | 1 |
|  | 2 | Cloud shadow | 0 |  |
|  | 3-5 | Land/water | 1 | Not 1 |
|  | 13 | Pixel adjacent to cloud | 0 |  |
| **QC** | 0-1 | MODLAND QA | 0 | >1 |
|  | 12 | Atmospheric correction | 1 | 0 |

**Supplementary Table 1.** Decision rules of the framework to attribute greening and browning to climatic or non-climatic factors based on the Theil-Sen (TS) slope of iNDVI, and the residuals resulting from the relationship between iNDVI and climate that indicates change in iNDVI not driven by climate.

| **#** | **iNDVI – climate relationship** | | **iNDVI**  **TS Slope** | **Assumption** | **Notes** |
| --- | --- | --- | --- | --- | --- |
|  | **Threshold** | **Residual TS Slope** |  |  |  |
| 1 | *P < 0.05* | Positive | Positive | Climatic  Greening | Positive iNDVI trend representing change due to climate and in the residuals representing change not due to climate. Variance of iNDVI signal explained by climate is greater than threshold indicates greening driven by climate |
| 2 | *P > 0.05* | Positive | Positive | Other  Greening | Same as #1 but variance of iNDVI signal explained by climate is lower than threshold indicates greening driven by other factors |
| 3 | *P < 0.05* | Negative | Positive | Climatic  Greening | Positive iNDVI trend representing change due to climate and negative residual trend representing change not due to climate. Variance of iNDVI signal explained by climate is greater than threshold indicates greening driven by climate |
| 4 | *P > 0.05* | Negative | Positive | Other  Greening | Same as #3 but variance of iNDVI signal explained by climate is lower than threshold indicates greening driven by other factors |
| 5 | *P < 0.05* | Negative | Negative | Climatic  Browning | Negative iNDVI trend representing change due to climate and in the residuals representing change not due to climate. Variance of iNDVI signal explained by climate is greater than threshold indicates browning driven by climate |
| 6 | *P > 0.05* | Negative | Negative | Other  Browning | Same as #1 but variance of iNDVI signal explained by climate is lower than threshold indicates browning driven by other factors |
| 7 | *P < 0.05* | Positive | Negative | Climatic  Browning | Negative iNDVI trend representing change due to climate and positive residual trend representing change not due to climate. Variance of iNDVI signal explained by climate is greater than threshold indicates browning driven by climate |
| 8 | *P > 0.05* | Positive | Negative | Other  Browning | Same as #7 but variance of iNDVI signal explained by climate is lower than threshold indicates browning driven by other factors |


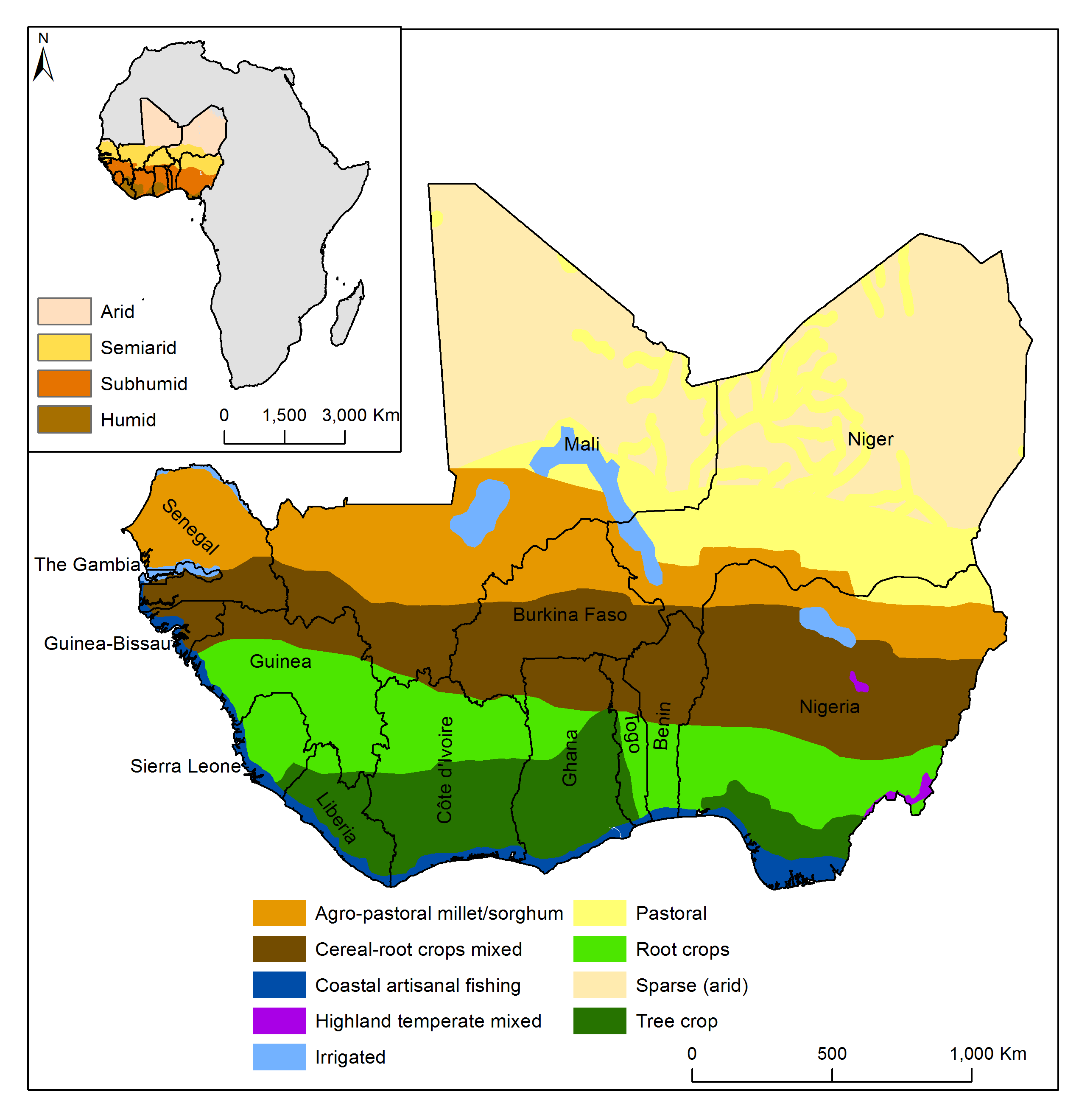


**Supplementary Fig. 1.** Africa-wide inset shows the location of the countries that form the Economic Community of West African States (ECOWAS) and their aridity zones. The main map shows the farming systems in the study area.


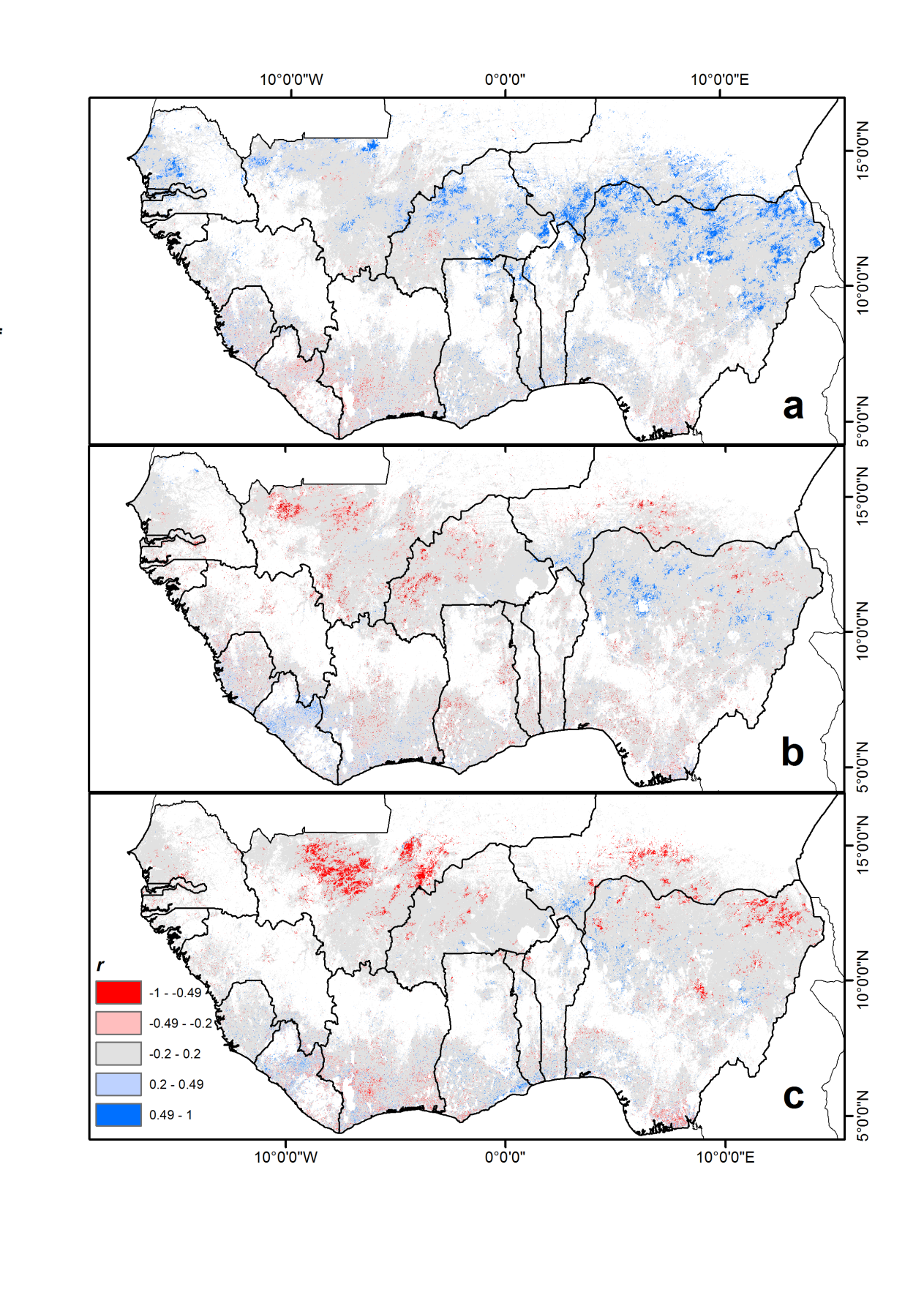


**Supplementary Fig. 2.** Spatial pattern of the partial correlation coefficients (r) of the relationship between iNDVI and (a) rainfall controlling for temperature and radiation, (b) temperature controlling for rainfall and radiation, and (c) radiation controlling for temperature and rainfall.


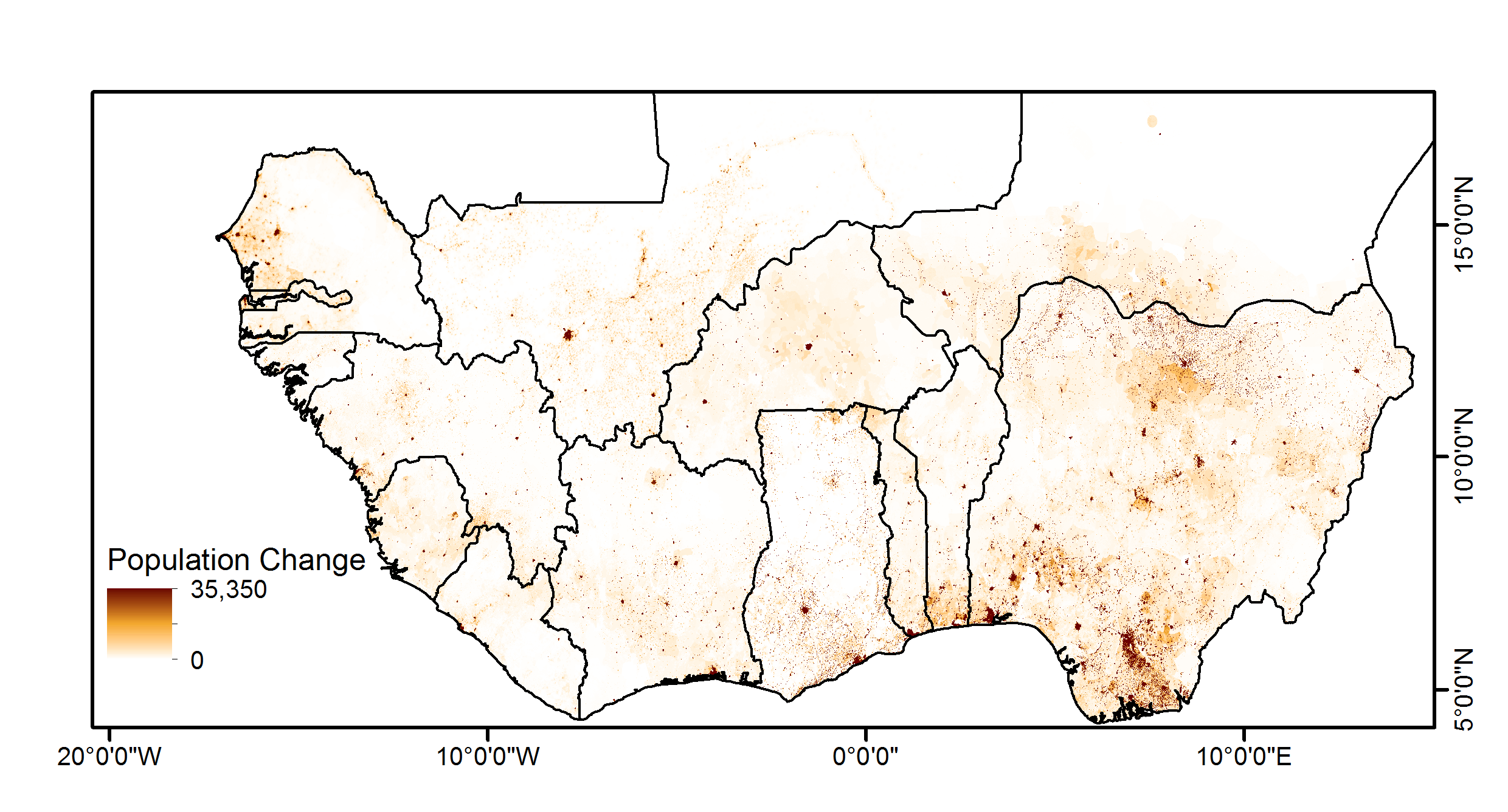


**Supplementary Fig. 3.** Human population trends between 2000 and 2015 at the 90% level.


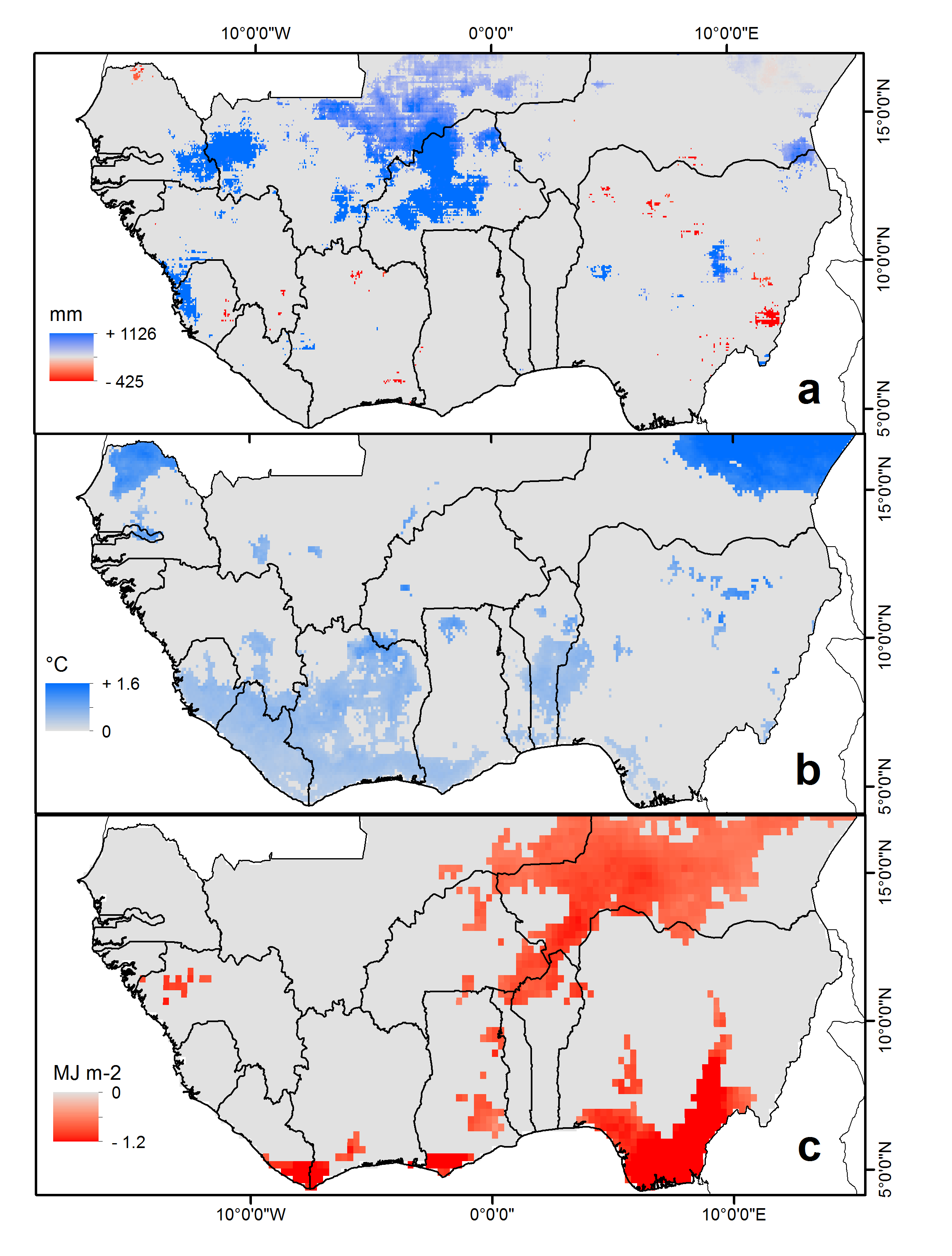


**Supplementary Fig. 4.** Significant trends between 2000 and 2018 for (a) total annual precipitation, (b) mean annual temperature, and (c) downward surface solar radiation.


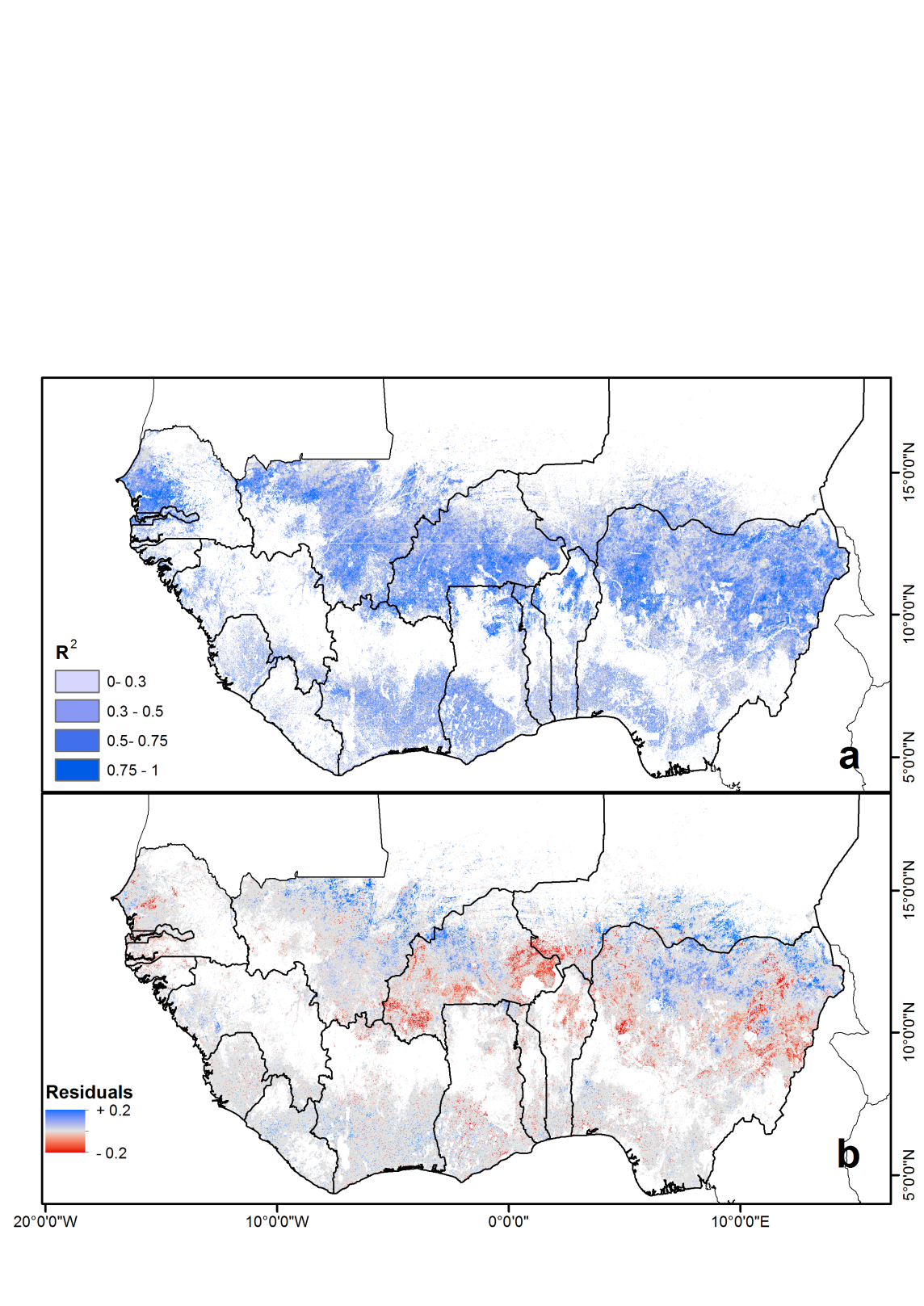


**Supplementary Fig. 5.** (a) Relationship between iNDVI and LOS for 2000 – 2018, and (b) trend of the residuals resulting from that relationship.


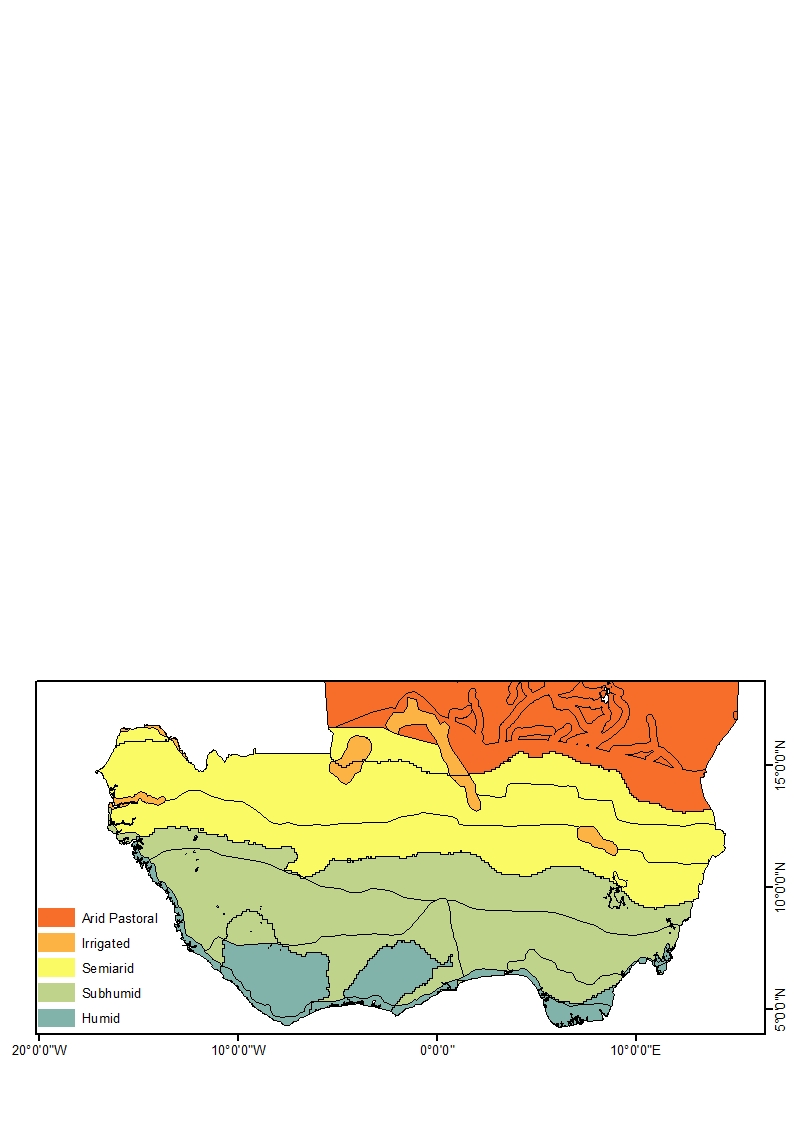


**Supplementary Fig. 6.** Merged moisture and farming system zones used for function fitting in TIMESAT.

1. Vermote, E. 2015. MOD09Q1 MODIS/Terra Surface Reflectance 8-Day L3 Global 250m SIN Grid V006.in N. E. L. P. DAAC, editor. [↑](#footnote-ref-2)
